# Influence of Fasting Plasma Glucose Level on Admission of COVID-19 Patients: A Retrospective Study

**DOI:** 10.1155/2022/7424748

**Published:** 2022-01-06

**Authors:** Yingying Zhao, Huichun Xing

**Affiliations:** Center of Liver Diseases Division 3, Beijing Ditan Hospital, Capital Medical University, Peking University Ditan Teaching Hospital, Beijing, 8 Jing Shun Dong Street, Beijing 100015, China

## Abstract

**Background:**

The coronavirus disease 2019 (COVID-19) is a serious global health threat and has spread dramatically worldwide. Prolonged viral shedding is associated with a more severe disease course and inflammatory reaction. Blood glucose levels were significantly associated with an increased hazard ratio (HR) for poor outcomes in COVID-19 patients.

**Objective:**

Previous studies focused primarily on the relationship between blood glucose and mortality or severe outcomes, but there were few research studies on the relationship between fasting plasma glucose (FPG) and duration of severe acute respiratory syndrome coronavirus 2 (SARS-CoV-2) RNA positive status. To explore the relationship between FPG levels and prolonged duration of SARS-CoV-2 viral positivity, the clinical data of COVID-19 patients were analyzed.

**Method:**

In this retrospective study, 99 cases of COVID-19 patients in Beijing Ditan Hospital were recruited, and their clinical and laboratory findings at admission were collected and analyzed. Furthermore, the risk factors for prolonged duration of SARS-CoV-2 RNA shedding were identified, and the relationship between FPG levels and the prolonged presence of SARS-CoV-2 RNA was evaluated.

**Result:**

We found that elevated FPG levels were correlated with longer duration of SARS-CoV-2 RNA positivity, classification of COVID-19, imaging changes of chest CT, inflammation-related biomarkers, and CD8^+^ T cell number in COVID-19 patients. In a logistic regression model, after adjusting for gender and age, COVID-19 patients with elevated FPG were more likely to had longer duration of SARS-CoV-2 RNA positivity than those with normal FPG levels (OR 3.053 [95% CI 1.343, 6.936]).

**Conclusion:**

Higher FPG levels (≥6.1 mmol/l) at admission was an independent predictor for prolonged SARS-CoV-2 shedding, regardless of a known history of diabetes. It suggests that intensive monitoring and control of blood glucose are important for all COVID-19 patients.

## 1. Introduction

The coronavirus disease 2019 (COVID-19) pandemic, which was caused by severe acute respiratory syndrome coronavirus 2 (SARS-CoV-2) infection, is a novel and serious global health threat and has dramatically spread worldwide [1]. COVID-19 is transmitted primarily through respiratory droplet and direct contact. At the time of this article's drafting, 188,616,093 confirmed cases and 4,065,804 deaths have been reported worldwide with new confirmed cases and deaths occurring per day [2].

Diabetes mellitus (DM) impacted outcomes of COVID-19 patients. A retrospective, single-center study in Iran showed that COVID-19 patients with DM had more comorbidities such as hypertension and complications than those without diabetes. Treatment failure and death were significantly higher in COVID-19 patients with diabetes compared to those without diabetes [3]. Serum levels of inflammation-related biomarkers such as IL-6, C-reactive protein, serum ferritin and coagulation index, and D-dimer were significantly higher in COVID-19 patients with DM compared with those without DM [4].

Fasting plasma glucose (FPG) level ≥ 7.0 mmol/l is one of the important criteria for the diagnosis of DM [5]. Regardless of a known history of DM, higher FPG levels significantly predicted mortality of COVID-19 (*P* < 0.05). Among nondiabetic COVID-19 patients, higher FPG remained a significant predictor of mortality [6, 7]. COVID-19 patients with an admission glucose level of >11 mmol/L were more likely to require intensive care unit (ICU), to develop acute respiratory distress syndrome (ARDS) and acute cardiac injury, and they had a higher death rate than patients with an admission glucose level of ≤11 mmol/L. In the multivariable analysis, COVID-19 patients with admission glucose level of >11 mmol/L had an increased risk of death and in-hospital complications, respectively [8].

Combined with some research results mentioned above, both persistent positivity of SARS-CoV-2 RNA and FPG affected outcomes of COVID-19 patients. Currently, there lacks research on the relationship between FPG and persistent positivity of SARS-CoV-2 RNA. We retrospectively analyzed the clinical data of COVID-19 patients in our hospital and further evaluated the relationship between FPG level and duration of SARS-CoV-2 RNA viral shedding/clearance. We tried to find the relevant factors affecting virus clearance and the possible factors that may influence the continuous positive status of SARS-CoV-2 RNA. These results will offer valuable information for the early control of COVID-19 in the real world and help reduce the social burden.

## 2. Materials and Methods

### 2.1. Study Design and Participants

All COVID-19 patients consecutively admitted to the hospital between June 1, 2020, and July 31, 2020, were collected. The diagnosis and clinical classification (mild, moderate, severe, and critical) of COVID-19 patients were carried out by two independent doctors based on the Guideline of Novel Coronavirus Pneumonia (8 th revised Edition) issued by the Chinese National Health Commission [6], mainly according to the criteria as follows: (1) patients with epidemiological history of novel coronavirus pneumonia (NCP), in contact with novel coronavirus infected people within 14 days prior to the onset of the disease; (2) any 2 of the clinical manifestations such as (i) fever and/or respiratory symptoms; (ii) the aforementioned imaging characteristics of NCP; (3) normal or decreased white blood cell count (WBC), and lymphocyte count in the early stage of onset; (4) positive real-time reverse transcription polymerase chain reaction (RT-PCR) results or highly homologous of viral gene sequence to known new coronaviruses [9].

**Figure 1:**
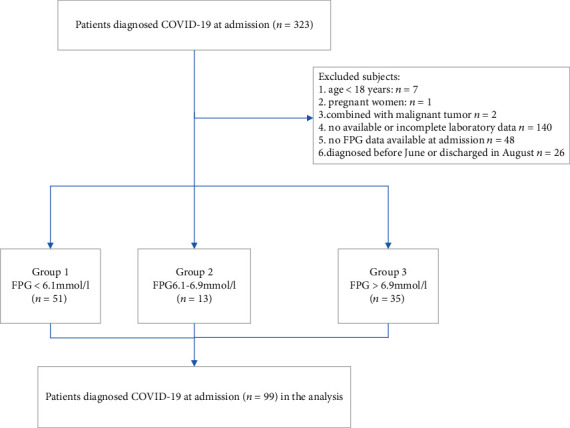
Flow diagram of patient selection.

Exclusion criterions included (1) age < 18 years, (2) pregnant women, (3) duplicated cases, (4) nonavailable or incomplete demographic or clinical data, (5) malignant tumor status, and (6) no FPG data available at admission (for example: FPG detected before admission or 24 h after admission, or routine blood glucose tests not being detected for each COVID-19 patient) (Figure 1).

All subjects were divided into 3 groups according to the FPG value at admission: group 1 (patients with FPG < 6.1 mmol/l), group 2 (patients with FPG 6.1 − 6.9 mmol/l), and group 3 (patients with FPG > 6.9 mmol/l).

The definitions and descriptions of some outcomes are as follows:

1. *Prolonged Viral RNA Shedding of SARS-CoV-2*. The negative conversion time (NCT) of SARS-CoV-2 RNA or the duration of SARS-CoV-2 RNA shedding was greater than the mean duration in the study
2. *Repositive or Recurrence of SARS-CoV-2*. SARS-CoV-2 nucleic acid was redetected in discharged patients.

### 2.2. Data Collection

FPG levels were measured at admission. For the test of FPG levels, blood samples were collected after an overnight fast lasting at least 8 h within 24 h after admission. The normal reference range of FPG in Beijing Ditan Hospital of Beijing is 3.9-6.1 mmol/L. To confirm SARS-CoV-2 infection, the real-time RT-PCR assay was used for detecting upper respiratory specimens (nasopharyngeal and oropharyngeal swabs), with or without a lower respiratory specimen (sputum). Nasopharyngeal swabs were collected on average every 3 to 7 days (serial time points).

We also obtained clinical, other laboratory, radiological, treatment, and outcome data from patients' electronic medical records for hospitalized patients. Past medical histories were obtained from hospital databases or through self-reporting, including type 2 diabetes mellitus (T2DM), hypertension, chronic lung disease, chronic heart disease, chronic liver disease, chronic kidney disease, cerebrovascular disease, and carcinoma, which were diagnosed according to standard criteria. The common complications that developed after hospitalization included acute respiratory distress syndrome (ARDS), acute cardiac injury, acute kidney injury, acute liver injury, cerebrovascular accident, coagulopathy, and secondary infection.

### 2.3. Statistical Analysis

All the patients completed the specimens collection and lung computed tomography (CT) scan within 48 hours after admission to ensure their diagnosis and classification of COVID-19. Continuous data were expressed as mean ± standard deviation (SD) or median (interquartile ranger, IQR). Categorical data were expressed as counts and proportions. The Kruskal-Wallis *H* test or Mann–Whitney *U* test was used to compare the differences between groups. Categorical variables were compared using the Chi-square (*χ*^2^) test or Fisher's exact test (if more than 20% of the cells had an expected count < 5), if appropriate. The data was analyzed through Spearman's bivariate correlations to evaluate the covariation between the duration of SARS-CoV-2RNA positive and FPG level or other indicators. Significant risk factors identified on univariate analyses were further analyzed by the multivariable logistic regression analysis to identify the independent risk factors associated with the prolonged duration of SARS-CoV-2 shedding, with adjustment for age, gender, and other potential confounding factors. Data were analyzed using SPSS 26.0 (IBM, Chicago, IL). For all the statistical analyses, *P* < 0.05 was considered significant.

## 3. Results

### 3.1. Characteristics of COVID-19 Patients at Admission

Among 323 diagnosed COVID-19 patients from June to July 2020, some patients were excluded for age < 18 years (*n* = 7), pregnant women (*n* = 1), combined with malignant tumor (*n* = 2), no available or incomplete laboratory data (*n* = 140), no FPG data available at admission (*n* = 48), and patients diagnosed before June or discharged in August (*n* = 26) were excluded. Finally, 99 cases were included in the study (Figure 1).

The mean age of the patients was 45.84 ± 12.87 years, and 54 patients (54.5%) were male. Forty-eight patients (48.5%) had FPG equal or higher than 6.1 mmol/L, and 51 patients (51.5%) had FPG < 6.1 mmol/L. Older patients were more prone to have elevated FPG (*P* < 0.05).

Among these patients, the most common symptom at onset of illness was fever [63 (63.6%)] (Table 1). Less common symptoms were cough (17 patients [17.2%]) and shortness of breath (11 patients [11.1%]). The proportions of sputum production (*n* = 8), muscle soreness (*n* = 6), and headache (*n* = 3) were 8.1%, 6.1%, and 3.0%. Of these patients, 15 patients (15.1%) had a history of diabetes. Other than diabetes, hypertension was the most common comorbidity (20patients [20.2%]) followed by chronic liver disease [6 patients (6.1%)]. The proportions of chronic lung disease, hyperlipidemia, and hyperuricemia were 5.1% (5 patients), 5.1% (5 patients), and 4% (4 patients), respectively.

The mean duration of SARS-CoV-2 RNA detection was 26.01 ± 6.71 days (Table 1). The shortest duration was 14 days, and the longest duration was 42 days. Those patients with higher FPG levels were also with a longer duration of viral persistence. There was a significant difference in the prolongation rate between the elevated FPG group and the normal FPG group (*χ*^2^ = 7.292, *P* = 0.007).

The proportions of SARS-CoV-2 RNA repositive in the three groups were not significantly different (*P* > 0.05) (Table 1). Recurrence of SARS-CoV-2 was correlated with past history of hypertension (*r* = 0.214, *P* = 0.033) (data not shown).

After stratification, the FPG levels in mild cases, moderate cases, and severe or critical cases were different (*P* < 0.05). Compared to mild cases, FPG levels were higher in moderate cases and severe or critical cases (*P* < 0.05) (Table 1).

Lung condition was assessed by chest CT scan. CT findings of lung involvement were significantly different among 3 groups (*χ*^2^ = 12.139, *P* = 0.009). Indicating patients were also with a higher FPG level accompanied by more severe CT chest findings (*r* = 0.296, *P* = 0.003).

We evaluated the proportion of major complications such as acute ARDS, liver injury, and secondary infection. The results showed that the percentages of patients with secondary infection were different in the 3 groups (*χ*^2^ = 10.486, *P* = 0.003). Compared with patients FPG < 6.1 mmol/l at admission, fever was not significantly related to longer viral RNA conversion time or the proportion of SARS-CoV-2 RNA repositive (*P* > 0.05) (Table 1).

### 3.2. Laboratory Findings

The analysis of laboratory parameters at admission showed that the levels of serum amyloid A (SAA), hemoglobin (HB), C-reactive protein(CRP), CD8^+^ T cell number, and erythrocyte sedimentation rate (ESR) were different among 3 groups (all *P* < 0.05) (Table 2). D-dimer was correlated with fever (*P* < 0.01). In addition, D-dimer was positively correlated with SAA (*r* = 0.253, *P* = 0.011), CRP (*r* = 0.238, *P* = 0.017), and ESR (*r* = 0.297, *P* = 0.003) (data not shown). While significantly negative correlations were showed between D-dimer and LYM (*r* = −0.231, *P* = 0.021), B cell (*r* = −0.200, *P* = 0.047), T cell (*r* = −0.204, *P* = 0.043), and CD8^+^T cell (*r* = −0.221, *P* = 0.028) (data not shown).

### 3.3. The Association between SARS-CoV-2 Related Indicators and Other Parameters at Admission

Spearman's correlation analysis showed that PDVPS was positively correlated with levels of FPG (*r* = 0.327, *P* = 0.001), different degrees of elevated FPG (*r* = 0.257, *P* = 0.010), FPG  ≥ ULN (*r* = 0.271, *P* = 0.007), D-dimer (*r* = 0.241, *P* = 0.016), and gender (*r* = 0.207, *P* = 0.040). And creatinine was negatively correlated with PDVPS (*r* = −0.208, *P* = 0.038).

Furthermore, levels of FPG (*r* = 0.281, *P* = 0.005), different degrees of elevated FPG (*r* = 0.245, *P* = 0.015), and FPG  ≥ ULN (*r* = 0.257, *P* = 0.010) were positively correlated with DSRP. In addition to elevated FPG related indicators, D-dimer (*r* = 0.215, *P* = 0.033), creatinine (*r* = −0.210, *P* = 0.037), and gender (*r* = 0.208, *P* = 0.039) were also correlated with DSRP (Table 3).

T2DM was related to severity of COVID-19 (*r* = 0.256, *P* = 0.011), fever (*r* = 0.212, *P* = 0.035), secondary infection (*r* = 0.288, *P* = 0.004), and imaging changes of chest CT (*r* = 0.257, *P* = 0.010) (data not shown). While T2DM was not significantly related to prolonged viral RNA conversion time or the recurrence proportion of SARS-CoV-2 RNA (*P* > 0.05) (Table 3).

The univariate logistic regression analysis suggested that creatinine (OR 0.972 [95% CI 0.946, 0.999]), gender (OR 2.326 [95% CI 1.036, 5.226]), FPG levels (OR 1.219 [95% CI 1.009, 1.474]), FPG ≥ 6.1 mmol/l (OR 3.053 [95% CI 1.343, 6.936]), and FPG grades (OR 1.756 [95% CI 1.124, 2.744]) were significantly associated with higher likelihood of PDVPS (Table 4). After adjusting for the gender, age, and diabetes history, the multivariable logistic regression analysis further suggested that FPG ≥ ULN (OR 3.053 [95% CI 1.343, 6.936]) was an independent predictor for PDVPS (Table 4). And the risk model was constructed as following: Logit (*P*) = -0.693 + 1.116 × (FPG ≥ ULN).

In addition, FPG was positively correlated with classification of COVID-19 (*r* = 0.393, *P* < 0.001), past DM history (*r* = 0.498, *P* < 0.001), SAA (*r* = 0.432, *P* < 0.001), CRP (*r* = 0.378, *P* < 0.001), ESR (*r* = 0.440, *P* < 0.001), and imaging changes of chest CT (*r* = 0.318, *P* = 0.001), while negatively correlated with CD8^+^ T cell number (*r* = −0.272, *P* = 0.006) (Table 5).

Similar to FPG levels, it showed consistent correlation between elevated FPG and DCC (*r* = 0.375, *P* < 0.001), past DM history (*r* = 0.436, *P* < 0.001), SAA (*r* = 0.332, *P* < 0.001), CRP (*r* = 0.326, *P* = 0.001), ESR (*r* = 0.366, *P* < 0.001), imaging changes of chest CT (*r* = 0.251, *P* = 0.012), and CD8^+^ T cell number (*r* = −0.252, *P* = 0.012) (Table 5).

## 4. Discussion

There have been few studies on the correlations between clinical features and the duration of SARS-CoV-2 RNA shedding. In our study, we evaluated the relationship between FPG level and PDVPS or DSRP, and identified factors that may affect the virus clearance and the prognosis of COVID-19.

We retrospectively analyzed the clinical data of COVID-19 patients in our hospital and further discussed the relationship between FPG levels and duration of SARS-CoV-2 RNA viral shedding or virus clearance. In our study, the mean duration of SARS-CoV-2 RNA viral shedding was 26.01 ± 6.71 days. In our cohort, patients with the longest viral shedding duration were 42 days. The range of duration SARS-CoV-2 RNA viral shedding was consistent with other studies, which ranged between 11 and 31 days [10].

The mean age of overall patients was 45.84 ± 12.87 years in our study. In this retrospective analysis, we found that DM patients with senior age and severe inflammatory characteristics of lung had increased incidences of comorbidities compared with those patients without diabetes. DM history was related to the severity of COVID-19 patients, fever, secondary infection, and imaging changes of chest CT (all *P* < 0.05). However, there was no significant relationship between preexisting diabetes or hypertension and the classification of COVID-19. Consistent with the study from Bennasrallah et al., diabetes and hypertension could not act as predisposing factors for late SARS-CoV-2 viral clearance [11]. In addition, T2DM was not significantly related to prolonged viral RNA conversion time or the proportion of RPV (*P* > 0.05). The possible reason was that although they had been diagnosed with diabetes before, their blood sugar levels were well controlled, which may be beneficial to themselves.

The Spearman's bivariate correlations showed that women were more likely to have long-term duration of SARS-CoV-2RNA positive. The univariate logistic regression analysis suggested gender was associated with a higher risk of PDVPS. Zhou et al. demonstrated that female sex was an independent predictor for prolonged SARS-CoV-2 RNA shedding [12]. While Xu et al. demonstrated that male sex was an independent predictor for prolonged SARS-CoV-2 RNA shedding [13]. Males and females might differ in immune reactivity [14]. However, these inconsistent results of the effect of gender on the duration of SARS-CoV-2 RNA shedding need to be further investigated in the follow-up research. Fu et al. found that D-dimer was related to the severity of COVID-19 [15]. However, we found that D-dimer was related to fever in this study. We also found that D-dimer was positively correlated with PDVPS and DSRP (*P* < 0.05). Moreover, D-dimer was positively related to inflammation-related biomarkers such as SAA, CRP, and ESR (all *P* < 0.05). Furthermore, D-dimer was negatively correlated to LYM, B cell, T cell, and CD8^+^T cell (all *P* < 0.05). It suggested the potential mechanisms were that D-dimer affected inflammation and immune responses.

Creatinine level was negatively related to PDVPS (*P* < 0.05). The univariate logistic regression analysis showed that creatinine was also associated with an increased risk of PDVPS. Low serum creatinine (SCr) could reflect to low skeletal muscle mass or sarcopenia and poor nutritional status [16, 17]. The condition of malnutrition could result in weaker cellular mediated immunity and impaired surfactant production [18, 19]. Therefore, patients with low serum creatinine may increase the risk of the inflammatory reaction in lung tissues of COVID-19 patients, which is related to PDVPS.

Although there was a correlation between FPG level and diabetes history in the study population, higher level of FPG was positively related to prolonged duration of SARS-CoV-2 clearance. The univariate logistic regression analysis suggested that FPG levels or FPG≥ULN were significantly associated with higher risk of PDVPS. The multivariate logistic regression analysis further indicated that FPG≥ULN was an independent predictor for PDVPS. There are some mechanisms indicated that higher FPG might play a role in the viral clearance of COVID-19 patients. Since immunity is the first line of defense against SARS-CoV-2, it appears that the disturbed immunity in patients is due to hyperglycemia. Hyperglycemia inhibits neutrophil chemotaxis, decreases phagocytosis by neutrophils, macrophages, and monocytes, and impairs innate cell-mediated immunity [20]. In the present study, the levels of T cell number and CD8^+^ T cell number were decreased. In addition, CD8^+^ T cell number was negatively correlated with FPG levels (*P* < 0.05). Moreover, serum levels of inflammation-related biomarkers such as SAA, CRP, and ESR were significantly elevated among COVID-19 patients with higher FPG (*P* < 0.05). Furthermore, after controlling for possible confounders, elevated FPG levels were related to the seriousness of COVID-19, accompanied by more severe CT chest findings and inflammation. And the probable mechanisms are that elevated FPG levels increase susceptibility to inflammation due to impaired T cell response, neutrophil function, and humoral immunity [21].

Recently, Zhao et al. found COVID-19 patients with hypertension had higher risk of recurrent detection of viral RNA by RT-PCR [22]. In the current study, we found that hypertension was associated with repositive of SARS-CoV-2 RNA (*P* < 0.05). The underlying mechanisms might be that hypertension can increase the expression of angiotensin converting enzyme-2 (ACE2). As is known to us, human cells that express ACE2 and transmembrane serine protease 2 (TMPRSS2) receptors act as portal of entry via direct interaction of the human body and immune system. The primary site of infection in COVID-19 is the upper and lower respiratory tract. There, SARS-CoV-2 infects goblet secretory cells of the nasal mucosa and alveolar type II pneumocytes by binding to membrane-bound ACE2, and further strengthening virus entry [23].

There were some limitations of our study. First, the interpretation of our results might be limited by the sample size. Second, owing to the retrospective design of the study, the lack of data did not allow us to analyze the mean in-hospital FPG. Third, because the number of critical cases was relatively small, the research on FPG among severe or critical cases may be limited in this study.

## 5. Conclusion

We found that higher FPG was an independent predictor of prolonged duration of SARS-CoV-2 RNA shedding/clearance in the present study. Our findings indicate that screening FPG level is an effective and simple method to evaluate the prognosis of patients with COVID-19, and intervention should be taken in time when patients with FPG ≥ 6.1 mmol/l regardless of a history of diabetes.

## Figures and Tables

**Table 1:** Characteristics of COVID-19 patients at admission.

Characteristic	All (*n* = 99)	Group 1 (*n* = 51)	Group 2 (*n* = 13)	Group 3 (*n* = 35)	*P* value
Age (years)	45.84 ± 12.87	41.1 ± 11.97	48.69 ± 12.34	51.68 ± 11.87	<0.001
Gender *n* (%)					0.106
Male	54 (54.5)	32 (32.3)	4 (4.0)	18 (18.2)	
Female	45 (45.5)	19 (19.2)	9 (9.1)	17 (17.2)	
DSRP days	26.01 ± 6.71	24.37 ± 6.20	27.85 ± 5.83	27.71 ± 7.27	0.042
PDVPS *n* (%)					0.026
Yes	46 (46.5)	17 (17.2)	8 (8.1)	21 (21.2)	
No	53 (53.5)	34 (34.3)	5 (5.1)	14 (14.1)	
DCC *n* (%)					0.002
Mild	19 (19.2)	17 (17.2)	0 (0)	2 (2.0)	
Moderate	72 (72.7)	32 (32.3)	12 (12.1)	28 (28.3)	
Severe/critical	8 (8.1)	2 (2.0)	1 (1.0)	5 (5.1)	
RPV *n* (%)					0.440
Yes	21 (21.2)	12 (12.1)	1 (1.0)	8 (8.1)	
No	78 (78.8)	39 (39.4)	12 (12.1)	27 (27.3)	
Temperature *n* (%)					0.321
<37.3 °C	36 (36.4)	22 (22.2)	5 (5.1)	9 (9.1)	
37.3-38°C	23 (23.2)	14 (14.1)	2 (2.0)	7 (7.1)	
38.1-39°C	26 (26.3)	11 (11.1)	4 (4.0)	11 (11.1)	
39.1-41°C	14 (14.1)	4 (4.0)	2 (2.0)	8 (8.1)	
Complications *n* (%)					0.873
Acute liver injury	35 (35.4)	16 (16.2)	5 (5.1)	14 (14.1)	0.691
Secondary infection	8 (8.1)	0 (0)	2 (2.0)	6 (6.1)	0.003
ARDS	7 (7.1)	1 (1.0)	1 (1.0)	5 (5.1)	0.089
T2DM *n* (%)	15 (15.2)	0 (0)	2 (2.0)	13 (13.1)	<0.001
Chest CT *n* (%)					0.009
No sign of pneumonia	6 (6.1)	6 (6.1)	0 (0)	0 (0)	
Unilateral pneumonia	21 (21.2)	13 (13.1)	5 (5.1)	3 (3.0)	
Bilateral pneumonia	72 (72.7)	32 (32.3)	8 (8.1)	32 (32.3)	

Note: group 1: FPG < 6.1 mmol/l; group 2: FPG 6.1 − 6.9 mmol/l; group 3: FPG > 6.9 mmol/l; Abbreviation: ∗RPV: repositive of virus, repositive of SARS-CoV-2 RNA; DSRP: duration of SARS-CoV-2 RNA positive; PDVPS: prolonged duration of SARS-CoV-2 RNA positive status; DCC: different classification of COVID-19. ∗*P* values were calculated by Kruskal-Wallis *H* test, Mann–Whitney *U* test, *χ*^2^ test, or Fisher's exact test, as appropriate.

**Table 2:** Laboratory examination of COVID-19 patients at admission according to different FPG levels.

	All (*n* = 99) (%)	Group 1 (*n* = 51)	Group 2 (*n* = 13)	Group 3 (*n* = 35)	*P* value
FPG (mmol/l)	5.96 (4.89, 7.35)	4.95 (4.51, 5.29)	6.34 (6.27, 6.56)	8.03 (7.24, 9.65)	<0.001
SAA (mg/l)	21.8 (3.2, 75.95)	11.4 (1.4, 38.7)	44.1 (2.4, 137.8)	49 (16.9, 141.15)	0.003
LAC (mmol/l)	2.98 (2.44, 3.65)	2.92 (2.27, 3.6)	2.78 (2.25, 3.6)	3.35 (2.52, 4.03)	0.261
BUN (mmol/l)	4.54 ± 1.20	4.54 ± 1.07	4.16 ± 1.36	4.70 ± 1.33	0.387
Crea (*μ*mol/l)	65.6 (56, 78.2)	66.9 (59.3, 78.9)	60.5 (52.4, 66.95)	62.6 (53.4, 79.6)	0.172
URCA (*μ*mol/l)	317 (256, 382)	343 (267, 417)	286 (220.5, 379)	303 (245, 358)	0.155
WBC (×10^9^/l)	5.12 ± 1.68	5.08 ± 1.63	5.30 ± 1.34	5.11 ± 1.89	0.916
LYM (×10^9^/l)	1.49 (1.1, 1.9)	1.63 (1.23, 2.09)	1.19 (1.09, 1.88)	1.37 (1, 1.82)	0.120
HB (g/l)	144 (130, 153)	148 (134, 160)	128 (119.5, 138)	143 (131, 149)	0.001
PLT (×10^9^/l)	193 (160, 235)	196 (171, 223)	212 (161.5, 251.5)	170 (144, 248)	0.337
ALT (U/L)	19.1 (13.6, 33.3)	19.9 (13.6, 32.7)	14.4 (11.5, 24.25)	19.6 (15.6, 35)	0.191
AST (U/L)	21.3 (17.1, 27.7)	19.5 (16.4, 27)	20.8 (17.7, 24.25)	22.7 (17.9, 30.3)	0.353
CRP (mg/l)	3.50 (0.8, 18.5)	1.7 (0.4, 6)	5 (1, 23.55)	9.1 (1.1, 35.5)	0.004
B cell number (cells/*μ*l)	171.00 (129, 259)	168 (142, 280)	148 (106.5, 233)	186 (122, 247)	0.485
NK cell number (cells/*μ*l)	217 (139, 313)	235 (150, 327)	228 (130.5, 311)	188 (139, 281)	0.379
T cell number (cells/*μ*l)	1004 (655, 1399)	1088 (846, 1497)	822 (540, 1220)	908 (612, 1314)	0.050
CD4^+^T cell number (cells/*μ*l)	583 (382, 825)	626 (493, 825)	395 (354, 899)	513 (373, 769)	0.159
CD8^+^T cell number (cells/*μ*l)	348 (220, 469)	396 (288, 483)	221 (146.5, 434)	290 (217, 420)	0.034
D-dimer (mg/l)	0.27 (0.14, 0.45)	0.25 (0.13, 0.42)	0.29 (0.20, 0.53)	0.3 (0.13, 0.45)	0.614
ESR (mm/h)	15 (7, 24)	10 (5, 19)	15 (12, 24.5)	20 (13, 40)	0.001

Note: group 1: FPG < 6.1 mmol/l; group 2: FPG 6.1 − 6.9 mmol/l; group 3: FPG > 6.9 mmol/l; abbreviation: ∗ALT: alanine aminotransferase; AST: aspartate aminotransferase; BUN: blood urea nitrogen; Crea: creatinine; UA: uric acid; LAC: lactic acid; SAA: serum amyloid A; ESR: erythrocyte sedimentation rate; WBC: white blood cell count; LYM: lymphocyte; NK cell: natural killer cell; Hb: hemoglobin; PLT: platelet; CRP: C-reactive protein. Data are mean, median (IQR), *n* (%), or *n*/*N* (%). ∗*P* values were calculated by Kruskal-Wallis *H* test, Mann–Whitney *U* test, *χ*^2^ test, or Fisher's exact test, as appropriate.

**Table 3:** Spearman's correlation between PDVPS/DSRP and other parameters.

Parameters	PDVPS		DSRP	
	Correlation	*P* value	Correlation	*P* value
RPV	-0.038	0.712	0.034	0.740
DCC	0.159	0.117	0.021	0.840
Degree of fever	0.158	0.118	0.087	0.392
Gender	0.207	0.040	0.208	0.039
T2DM history	0.171	0.090	0.164	0.105
FPG grades	0.257	0.010	0.245	0.015
FPG ≥ ULN	0.271	0.007	0.257	0.010
FPG mmol/l	0.327	0.001	0.281	0.005
Crea *μ*mol/l	-0.208	0.038	-0.210	0.037
CD8^+^ T cell number cells/*μ*l	-0.239	0.017	-0.191	0.058
D-dimer	0.241	0.016	0.215	0.033

Abbreviation: ∗PDVPS: prolonged duration of SARS-CoV-2 RNA positive status; ULN: upper limit of normal; RPV: repositive of virus, repositive of SARS-CoV-2 RNA; DCC: different classification of COVID-19; FPG grades: different groups of FPG levels, including <6.1 mmol/l, 6.1-6.9 mmol/l, and >6.9 mmol/l; Crea: creatinine.

**Table 4:** Bivariate logistic regression of the association between clinical parameters and PDVPS.

Parameters	PDVPS	
	OR (95% CI)	*P* value
FPG mmol/l	1.219 (1.009, 1.474)	0.041
FPG ≥ ULN	3.053 (1.343, 6.936)	0.008
FPG grades	1.756 (1.124, 2.744)	0.013
Gender		
Male		
Female	2.326 (1.036, 5.226)	0.041
Crea *μ*mol/l	0.972 (0.946, 0.999)	0.041

Abbreviation: ∗PDVPS: prolonged duration of SARS-CoV-2 RNA positive status; ULN: upper limit of normal; FPG grades: different groups of FPG levels, including <6.1 mmol/l, 6.1-6.9 mmol/l, and >6.9 mmol/l; Crea: creatinine.

**Table 5:** Spearman's correlation between FPG and other parameters.

Parameters	FPG		FPG ≥ ULN	
	Correlation	*P* value	Correlation (95% CI)	*P* value
DCC	0.393	<0.001	0.375	<0.001
Degree of fever	0.252	0.012	0.224	0.026
Chest CT	0.318	0.001	0.251	0.012
CD8^+^ T cell number cell/*μ*l	-0.272	0.006	-0.252	0.012
ESR mm/h	0.440	<0.001	0.366	<0.001
CRP mg/l	0.378	<0.001	0.326	0.001
SAA mg/l	0.432	<0.001	0.332	0.001

Abbreviation: ∗ULN: upper limit of normal; DCC: different classification of COVID-19; ESR: erythrocyte sedimentation rate; CRP: C-reactive protein; SAA: serum amyloid A.

## Data Availability

The clinical data used to support the findings of this study are included within the article.
